# Predicting Perceived Health of Older Adults: The Role of Health, Personality, and Resilience

**DOI:** 10.1093/geroni/igaa057.734

**Published:** 2020-12-16

**Authors:** Rotem Arieli, Joseph Kim, Peter Martin

**Affiliations:** Iowa State University, Ames, Iowa, United States

## Abstract

Past research has not addressed how domain-specific “health” personality traits are associated with resilience and well-being. The purpose of this study was to determine pathways from health personality to perceived health, mediated by resilience. Data included 3,907 participants, 65 and older, collected by a large provider of Medicare Supplemental Health Insurance. The Health Personality Assessment (health neuroticism, health extraversion, health openness, health agreeableness, and health conscientiousness), Brief Resilience Scale, and perceived health were measured. Structural equation modeling and bootstrap mediation were conducted in Mplus. The hypothesized model resulted in a marginal fit, so direct paths from health openness and health conscientiousness to perceived health were added, resulting in an improved fit, χ2(192)=1660.96, RMSEA=.04, CFI=.95; χ2∆(2)=403.99, p<.001. Health neuroticism and health extraversion negatively predicted perceived health, fully mediated by resilience, β=-.11, p<.001, and β=-.01, p<.05, suggesting that people anxious about their health or that talk about their health had significantly lower levels of resilience. Resilience positively predicted perceived health, indicating that more resilient people reported better health. Higher levels of health openness predicted significantly lower levels of perceived health, β=-.19, p<.001. Greater levels of health conscientiousness predicted better perceived health, β=.20, p<.001, and resilience in-turn positively related to perceived health, β=.08, p<.001. Health personality and resilience explained 25.3% of variance in perceived health. This study exemplifies the importance of health personality and resilience in predicting perceived health for older adults. Future research should examine interventions focused on health personality increasing resilience, as older adults with higher resilience reported significantly better health.

